# Student usage of open educational resources and social media at a Sri Lanka Medical School

**DOI:** 10.1186/s12909-022-03106-2

**Published:** 2022-01-13

**Authors:** Samankumara Hettige, Eshani Dasanayaka, Dileepa Senajith Ediriweera

**Affiliations:** Health Data Science Unit, Faculty of Medicine, University of Kelaniya, Ragama, Sri Lanka

**Keywords:** Free educational resources, Academic information seeking, Medical students, Open educational resources, Social media, Wiki, Facebook

## Abstract

**Background:**

The use of Open Educational Resources (OER) and Social Media (SM) for academic information seeking is common among undergraduates nowadays. There is limited data on OER and SM use for education in Sri Lanka. This study was aimed at evaluating the OER and SM use for education among the medical students at the Faculty of Medicine, University of Kelaniya, Sri Lanka.

**Methods:**

A cross-sectional study was conducted at the Faculty of Medicine, University of Kelaniya. Stratified random sampling was used to select students from the first year to the final year. A self-administrated questionnaire was used to collect data.

**Results:**

The study included 257 responses (response rate: 89.5%), of which 185 (72.0%) were females. The OER and SM use for educational purposes at least once a month among students was 96.1% (95%CI: 93.7–98.5%) and 88.3% (95%CI: 84.4–92.3%) respectively. There was no gender difference in OER and SM use. The main reasons for accessing OER were the availability of information at any time (36.1%) and ease of information access (31.5%). Wiki sites (84.4%) and Facebook (79.8%) were the highest accessed OER and SM platforms. The majority of students were in view that the information on wiki sites (51.4%) and results of general non-specific web searches (56.0%) were reliable. Only 33.9% of students searched information from educational and government-related sources and 18.7% had accessed e-journals. Through SM, 79.0% joined educational groups and 77.0% followed the medical-related sites, pages and people. More than one-third of students (35.8%) could not find academic information from SM due to the information overload and 31.1% mentioned that SM distracted their education.

**Conclusion:**

The majority of the students used OER and SM for education; however, only a minority accessed reliable information. Students accepted information available in wiki sites and general non-specific web searchers without considering the credibility of sources. The majority of the students did not refer to e-journals. Distractions to academic work and the difficulty to access accurate information were major concerns of using SM. This study highlights the importance of improving information literacy among medical students.

**Supplementary Information:**

The online version contains supplementary material available at 10.1186/s12909-022-03106-2.

## Introduction

Web 1.0 technology was invented in the mid-1990s enabling users to search and read the content available on the Web [1]. Web 2.0 technology was launched in 1999 facilitating users to read and write content on the Web. Technological advancements paved the way to collaborative writing (e.g. Wikipedia, Harvard Health Blog), content sharing (e.g. text, video, and images) and social networking (e.g. Facebook, Twitter) [2, 3]. Open Educational Resources (OER) are the teaching and learning resources that are freely available on the Web. These include online courses, course materials, textbooks, videos, examination questions, mock tests, software and any other tool that support access to knowledge [4]. The group of applications that allow the creation and exchange of User Generated Content on the Web are defined as Social Media (SM) [5].

The OpenCourseWare project is considered a milestone in the OER era. The project was initiated by Massachusetts Institute of Technology where the educational materials were made freely available for users [6]. This initiative drew the attention of educators to develop OER around the world [7]. The majority of OER was developed in Europe and North America at the beginning of the twenty-first century, and the contribution to OER from Asia was less than 5% [8–10]. In 2002, UNESCO’s Open Courseware Forum helped to formalize the OER and facilitated lifelong learning opportunities for learners [6, 11, 12]. The “Paris Declaration” at the first OER World Congress in 2012 and “Ljubljana Declaration” at the second OER World Congress in 2017, further promoted OER usage around the world [13]. OER has changed the way how students learn and institutions operate [12]. OER has the potential to transform the teaching and learning process [14, 15]. By now, both developed and underdeveloped countries use the OER in education systems [11].

The combination of mobile device and web-based technologies have produced interactive SM platforms through which communities jointly create, share, discuss and modify content [5] [16]. The positive impact of SM on education has been highlighted in the literature [17–20].

### Terminology

#### Open educational resources

Open Educational Resources (OER) are teaching, learning and research materials in any medium – digital or otherwise – that reside in the public domain or have been released under an open license that permits no-cost access, use, adaptation and redistribution by others with no or limited restrictions. OER form part of ‘Open Solutions’, alongside Free and Open Source software (FOSS), Open Access (OA), Open Data (OD) and crowdsourcing platforms [21].

The common OER include Wiki sites, Blogs, SlideShare, e-Journals and bibliographic databases. A wiki is a website built with facilities for users to add and update content on the site using their web browsers [22]. A blog is an online journal or informational website that contains dated entries (posts) in reverse chronological order, so that the most recent post appears first, at the top. It is a platform where one person or a group of contributors can participate in sharing their views on a particular topic [23, 24]. SlideShare is the world’s largest repository of PowerPoint presentations on various topics [25]. An e-Journal is a periodical publication that is solely published on the Web and in a digital format [26]. A bibliographic database is a database of bibliographic records, an organized collection of references to published digital literature [27].

#### Social media platforms

Social Media (SM) are Internet and mobile-based tools and devices that integrate technology, telecommunications, and social interaction enabling the construction, co-construction and dissemination of words, images (static and moving) and audio [28].

Facebook, YouTube and Twitter are examples of the most commonly used SM platforms in today’s world. Facebook is a social network site initially designed to support a college network at Harvard University. Now anybody can join Facebook via the Internet and create a member profile. Users can personalize the appearance of their profile, post videos and photos on their homepages, invite people to be their friends and accept friendship invitations from others [28]. YouTube is a video-sharing website that enables the user to watch videos and post the user’s videos to share with others [29]. Twitter is a microblogging site that enables users to exchange very short messages [29]. Google+ is another example of a social network site, owned and operated by Google [30].

#### Connectivism learning theory

Connectivism is a prominent network learning theory, developed for e-learning environments [31]. The founder, George Siemens introduced it as a learning theory for the digital age. One underlying assumption in this theory is that knowledge is distributed across an information network or multiple individuals and can reside outside of ourselves (within an organization or a database). The starting point for learning occurs when knowledge is actuated through the process of a learner connecting to and feeding information into a learning community. A learning community is defined as a group of people learning together through continuous dialogue because of their similar interests that allow for interaction, sharing, dialoguing and thinking together [32].

Connectivism is driven by the understanding that any decision taken at one point in time may change due to rapidly altering foundations. New information is continuously being generated and acquired. Connectivism highlights the abilities to find the latest information and to filter the information to differentiate between important and unimportant information as important skills in the learning process. The ability to make decisions on the information is a cornerstone of the learning process [32]. Far reaching use of the Internet in today’s world paves the path for the applications of connectivism theory for learning [33].

### The theoretical underpinnings and conceptual framework

This study aimed at investigating the extent to which medical students use OER and SM for academic purposes. Connectivism learning theory explains how Internet technologies have created new opportunities for people to learn and share information across the World Wide Web and among themselves [32]. Hence, a conceptual framework based on the principles of the theory of connectivism was employed in the present study.

The following theoretical and practical aspects were concerned with the formation of study objectives.

- The information on the Web changes quickly and the ability to identify meaningful information is an essential skill in connectivism learning theory [29] [32]. This skill is related to the Information Literacy (IL) of students. Previous literature shows medical students lag behind the required level of IL [34, 35], particularly in developing countries including Sri Lanka [36].
- Connectivism asserts that learning happens through connections and, continuous knowledge growth requires continuous networking and nurturing network connections [32]. In that sense, SM facilitates more user interactions which help to nurture the relationships between people [37].
- The Faculty of Medicine, University of Kelaniya provides IT infrastructure for students where Internet facility is readily available for them. This allows students to maintain uninterrupted network connections to continue learning without any hindrance as specified in connectivism. Apart from the network connectivity, the students may confront the other factors which affect OER and SM use in education.

### Study goals

The study aimed at evaluating the use of OER and SM for academic purposes among the medical students at the Faculty of Medicine, University of Kelaniya, Sri Lanka.

Therefore, the study is guided by the following objectives.

- ° Identify the OER and SM platforms utilized by the medical students for academic purposes.
- ° Find the extent to which such resources and platforms are utilized.
- ° Examine the advantages of utilizing such resources and platforms for academic purposes.
- ° Find the limitations faced by students in the use of OER and SM for education.
- ° Determine the reliability of academic information extracted via OER and SM platforms.
- ° Determine the SM interactive features which attract students in pursuit of education.

The outcome of this study contributes to the existing literature in which much evidence cannot be found to convince the availability of data on OER [13, 38] and SM [39, 40] for academic purposes in medical education, mainly by students in developing countries. The finding of the study will also be useful for academics, administrators and policymakers in higher education institutions, especially in developing countries and particularly in Sri Lanka when considering the integration of modern techniques and technologies in the learning process.

## Methodology

A cross-sectional study was undertaken at the Faculty of Medicine, University of Kelaniya, Sri Lanka.

### Student background and institutional context

Sri Lanka offers free education at all levels, including tertiary education. Students are free to apply for any Medical Faculty in Sri Lanka if they fulfil the minimum requirements in the admission criterion. A selection process is carried out by the University Grants Commission of Sri Lanka to allocate students to Medical Faculties based on their GCE Advanced Level examination performance and their preference of universities.

### Educational context

The Faculty of Medicine, University of Kelaniya is listed in the World Health Organization Directory of Medical Schools and its MBBS degree is recognized by the General Medical Council of the United Kingdom [41]. The Faculty offers a five-year MBBS course consisting of three 10-week terms each year. The Faculty has adopted an organ-system based integrated curriculum for its MBBS degree. The clinical training commences from the third year where students undergo clinical attachments during the third and fourth years along with the coursework. A full-time hospital-based clinical learning takes place in the final year where students undergo 2 months of training in Medicine, Surgery, Obstetrics & Gynecology, Pediatrics and Psychiatry. There are three main examinations in the MBBS program which take place at the end of the second, fourth and final years. Graduates must complete a 12 month internship period at a government hospital to obtain full registration to practice medicine in Sri Lanka [41].

### Population and sampling

This study was conducted from August 2016 to December 2016 at the Faculty of Medicine, University of Kelaniya. There was a total of 903 students in the Faculty by then. A stratified random sampling method with proportional allocation was used to recruit 287 students for the study. Table 1 provides the allocated number of students from each batch. Name lists of the students from each batch were obtained and students were selected using a random number generator (sample() function in R statistical package). The detailed procedure of sample size calculation is included in supplementary material 1.

**Table 1:** Allocated number of students from each batch

Academic year	Total number of students	Selected number of students
First-year	163	52
Second-year	174	55
Third-year	168	54
Fourth-year	199	63
Final-year	199	63
Total	903	287

### The study instrument

A self-administered questionnaire was given to students. The questionnaire consisted of two components and 21 items including academic year, gender and interest in post-graduate studies and questions on OER and SM usage, frequency, reasons for usage and limitations. The questionnaire was developed and designed by the authors based on a similar study done in Sri Lanka [42]. Questions were modified with expert opinions considering the local and cultural settings to ensure content validity (supplementary material 2). The questionnaire was piloted and updated according to the suggestions made by the Ethical review committee.

Ethical approval for the study was obtained from the Faculty of Medicine, University of Kelaniya (protocol number – FWA00013225). The research was carried out as per the Declaration of Helsinki. At least one of the authors participated in the data collection process. All the medical students were over 18 years of age. All the students were given information sheets and informed written consent was obtained after explaining the details of the study. As all the participants are above 18 years of age, the consent of parents or legal guardians was not necessary.

### Data analysis

The identified data were entered into a password-protected database and only investigators had access to the data. The statistical analysis was done using R version 3.5.3. The average and daily usage of OER and SM were calculated with confidence intervals. Descriptive statistics were calculated for each OER, SM platforms and other variables. Chi-square and Fisher’s exact test statistics were used to check the statistical differences with usage differentiating according to gender, academic year and other variables appropriately. *P* < 0.05 was considered statistically significant.

## Results

Of 287 distributed questionnaires, (89.5%) responses were received, and all these responses were considered for the analysis. There were 185 (72.0%) female students. The number of students who responded to the questionnaire from the first year to the final year with the percentage for each year is as follows: 52 (20.2%), 55 (21.4%), 48 (18.7%), 57 (22.2%) and 45 (17.5%).

### The usage of OER for academic purposes

Among respondents, 247 (96.1%; 95%CI: 93.7–98.5%) used OER for academic purposes. The usage was not significantly different between males and females (86.1% vs 97.3% respectively; *P* = 0.2). OER usage from first to final year was 51 (98.1%), 53 (96.4%), 44 (91.7%), 54 (94.7%) and 45 (100%) respectively. The usage did not show a significant difference between the year of study (*P* = 0.2). OER usage was not different among students who had different post-graduate study plans (Yes- 113 (98.3%), No- 25 (96.2%), Not decided- 100 (94.3%), *P* = 0.2). There were 131 (51.0%; 95%CI: 44.9–57.1%) students who daily used OER (Table 2).

**Table 2:** OER and SM usage with different categories

Category	Open Educational Resources (OER)	***P*** value	Social Media (SM)	***P*** value
Usage *	247(96.1%)		227(88.3%)	< 0.01
Daily usage ^†^	131(51.0%)		175(68.1%)	< 0.001
Usage vs Gender				
Males (72)	62 (86.1%)	0.2	62 (86.1%)	0.6
Females (185)	180 (97.3%)		165 (89.2%)	
Usage vs post-graduate planning				
Yes (115)	113 (98.3%)	0.2	102 (88.7%)	0.07
No (26)	25 (96.2%)		20 (77.0%)	
Not-decided (106)	100 (94.3%)		98 (92.5%)	
Usage vs Academic years				
First year (52)	51 (98.1%)	0.2	49 (94.2%)	0.08
Second year (55)	53 (96.4%)		52 (94.5%)	
Third year (48)	44 (91.7%)		39 (81.3%)	
Fourth year (57)	54 (94.7%)		47 (82.5%)	
Final year (fifth year) (45)	45 (100%)		40 (88.9%)	

*Highly significant difference (*P* < 0.01) between OER and SM usage was noted; †Very highly significant difference (*P* < 0.001) was found between SM and OER daily usage

Wiki sites were the most popular OER (84.4%) among students followed by SlideShare (34.2%). Only 18.7% accessed e-journals and 5.8% used the e-journal facility offered by the Faculty. The main reasons to use OER were the availability of information at any time (36.1%), ease of searching relevant information (31.5%), availability of detailed information (16.0%) and up-to-date information (7.0%). Reading digital screens for longer periods was the main obstacle (40.9%) and 3.5% had limited access to OER (Table 3).

**Table 3:** The popular Open Educational Resources (OER) among the students and reasons to use them in education and limiting factors of using OER

Resource	Number	Percentage (%)
Wikis	217	84.4
SlideShare	88	34.2
e-journals	48	18.7
Bibliographic databases	25	9.7
Faculty e-journal collection	15	5.8
Medical forums	9	3.5
Reasons to seek information		
Availability	93	36.1
Easy to search	81	31.5
Detailed information	41	16.0
Updated information	18	7.0
Limiting factors		
Difficulty to read the screen for a longer period	105	40.9
Lack of time during the academic hours	68	26.5
Distrustful content in OER	20	7.8
Information overload	19	7.4
Lack of facilities (PC/Laptop/smart phone and Internet)	9	3.5

**Fig. 1:**
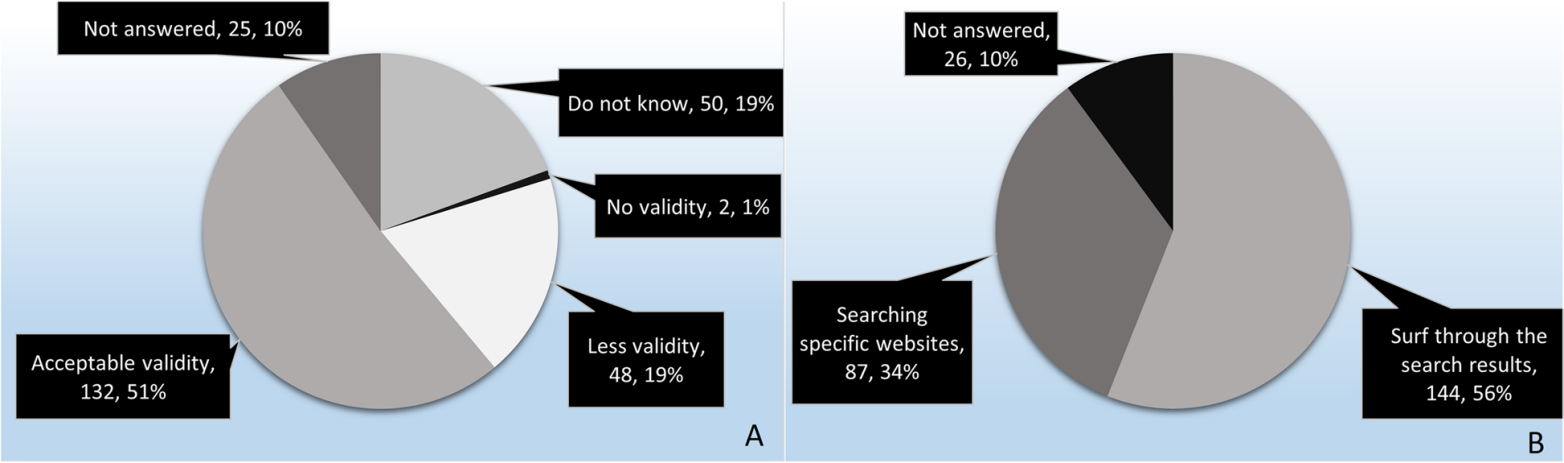
Students’ perception about information validity on wikis and web searches

Among the wiki site users, 132 (51.4% of total) were of the view that the information available on these sites was reliable (Fig. 1A). There were 231 (89.9%) students who used general web searches to find academic information. Among them, 144 (56.0% of total) relied on the information found in general web searches without considering the creditability of the source and only 87 (33.9% of total) specifically searched information in educational and government-related sources (Fig. 1B).

### The usage of SM for academic purposes

Among the participants, 227 (88.3%; 95%CI: 84.4–92.3%) used SM for academic purposes. Male and female students did not show a difference in SM usage (86.1% vs 89.2% respectively; *P* = 0.6). The SM usage from first to final year was 49 (94.2%), 52 (94.5%), 39 (81.3%), 47 (82.5%) and 40 (88.9%), which was not significantly different (*P* = 0.08). The usage did not show a difference with respect to post-graduate plans of students (Yes-102 (88.7%), No-20 (77.0%), Not decided-98 (92.5%), *P* = 0.07). There were 175 (68.1%; 95%CI: 62.4–73.8%) students who daily accessed SM. The number of daily users was higher in SM compared to OER (*P* < 0.001) (Table 2).

Facebook (79.8%) was the most popular SM platform among the participants followed by YouTube (60.3%) and Google+ 129 (50.2%). The number of students who had never used Facebook, YouTube and Google + for educational activities were 12 (4.7%), 7 (2.7%) and 24 (9.3%) respectively (Table 4).

**Table 4:** The status of four social media platforms used in education by medical students

Social media	Usage Number (%)	Daily	Once a week	Once a month	Never	Not answered
Facebook	205 (79.8%)	146 (56.8%)	46 (17.9%)	13 (5.1%)	12 (4.7%)	40 (15.5%)
You Tube	155 (60.3%)	55 (21.4%)	76 (29.6%)	24 (9.3%)	7 (2.7%)	95 (36.9%)
Google +	129 (50.2%)	68 (26.5%)	40 (15.6%)	21 (8.2%)	24 (9.3%)	104 (40.4%)
Twitter	46 (17.9%)	17 (6.6%)	18 (7.0%)	11 (4.3%)	41 (16.0%)	170 (66.1%)

Two hundred and three (79.0%) students participated in educational groups within SM and 77.0% followed medical-related sites, pages and people. One hundred and six (41.2%) students involved in academic discussions and sharing information with foreign students through SM. The main factors restraining students using SM were the overload of unwanted information (35.8%) and disturbance to academic work (31.1%) (Table 5).

**Table 5:** Popular academic activities via SM and limitations of using SM in education

	Number	Percentage (%)
Academic activities through SM		
Participation in educational groups	203	79.0
Following medical-related sites, pages and people	198	77.0
Academic discussions and sharing information	106	41.2
Limitations of using SM		
Excess of unwanted information	92	35.8
Affect to the concentration on academic activities	80	31.1
Distrust of information shared by others	32	12.5
Lack of facilities (PC/Laptop/smart phone and Internet)	6	02.3

The popular SM activities and educational involvement related to gender and academic years are illustrated in Table 6. There was no difference in participation in educational groups and the following medical-related sites, pages and people based on gender or academic year. Students’ involvement in academic discussions and sharing information with foreign students through SM were significantly different between academic years (*P* = 0.004), where the highest involvement was among the first-year students (61.5, 95%CI: 55.6–67.5%) and the lowest among the final year students (22.2, 95%CI: 17.1–27.3%) (Table 6).

**Table 6:** Popular Social Media (SM) activities and educational involvement related to gender and academic year

Category	Social Media (SM)	***P*** value
Joining the educational groups vs Academic years		
First year	43 (82.7%)	0.7
Second year	44 (80.0%)	
Third year	36 (75.0%)	
Fourth year	41 (71.9%)	
Final year (fifth year)	39 (86.7%)	
Joining the educational groups vs Gender		
Males	52 (72.2%)	0.3
Females	151 (81.6%)	
Following medical-related sites, pages and people vs Academic years		
First year	43 (82.7%)	0.2
Second year	45 (81.8%)	
Third year	30 (62.5%)	
Fourth year	42 (73.6%)	
Final year (fifth year)	38 (84.4%)	
Following medical-related sites, pages and people vs Gender		
Males	56 (77.8%)	0.5
Females	142 (76.6%)	
Academic discussions and sharing information vs Academic years		
First year	32 (61.5%)	0.004
Second year	25 (45.5%)	
Third year	19 (39.6%)	
Fourth year	20 (35.1%)	
Final year (fifth year)	10 (22.2%)	
Academic discussions and sharing information vs Gender		
Males	34 (47.2%)	0.1
Females	72 (39.0%)	

Number of students: First year = 52, Second year = 55, Third year = 48, Fourth year = 57, Final year = 45, Males = 72, Females = 185

## Discussion

### OER usage

Data pertaining to OER here displayed a high OER usage (96%) amongst students (Table 2). These students do not have access to academic or government databases and their main sources of knowledge are textbooks, lecture notes and student-teacher interactions. Therefore, OER can be considered as an alternative educational method in the local context and this highlights the importance of facilitating OER in medical education in Sri Lanka. OER access among the participating students in our study was higher than the reported participation in previous studies in Zhejiang University in China [38] and the University of Leeds in the United Kingdom [43]. OER usage differences between the year of study or genders cannot be seen in the present study (Table 2). Similar findings were reported in Australia [44].

Wiki sites were the most popular OER (84%) among the students (Table 3). This was different to a previous study done in China where 60% of students used video materials and only 45% referred text related resources [38]. The contents in Wiki sites are difficult to verify due to lack of references. The contents in these sites can be subjected to the motives of editors and the public as they can edit the content [45, 46]. However, the majority of the students (more than 51%) were of the view that the information on wiki sites was accurate (Fig. 1) [45, 47]. Students’ unawareness of how to obtain reliable information from web-based resources reflects deficiency in IL. Therefore, this study highlights the importance of incorporating components on IL in undergraduate medical curricula [48, 49].

SlideShare was the second most popular OER (Table 3) and students frequently accessed this repository to download educational materials. SlideShare may contain unverified educational materials. However it allows users to search and filter out materials by authors [50]. Such features are not available on all OER platforms; therefore, users need to have a basic understanding of these different platforms to assess the reliability of their contents. For instance, Wiki sites allow anonymous entries and make it difficult to find the identity of the editor compared to the contents on SlideShare, where users can search for materials by the author.

The e-journals are considered as valuable OER in medicine [51]. However, medical students in our study showed a lower preference to access e-journals (Table 3). Around 6% of students used the e-journal facility provided by the Faculty. This could be due to the lack of awareness or motivation to discover new knowledge [52–54]. Encouraging students to use e-journals is important to facilitate student research activities and to foster high-quality medical education [55, 56].

A key feature of any e-learning platform is the availability of materials for users at the fingertip [57]. Our study also indicates that the main reasons for students to use OER are the availability of information at any time and the ease of access from any place. However, only a minority accessed OER to obtain detailed (16%) and up-to-date (7%) information (Table 3).

### Perception of quality of OER

There are abundant OER on the Internet including unreliable materials (e.g. Wikis, File repositories) as well as good quality materials developed by reputed organizations with well-defined teaching aims [50]. Students should be able to differentiate good and bad quality OER and assess the creditability of the source.

Our study indicates that the participating medical students can conduct general web searches for information gathering. However, the use of reliable and good quality materials from web searches was low (Fig. 1B). We gave two options in the questionnaire (given below) to understand how students conducted general web searches.

- Option 1: Looking for results without considering the site (e.g. surf through the results).
- Option 2: Searching specific sites (e.g. .edu, .gov, nih.gov).

More than 60% of respondents selected option one. It appeared that most of the students (56% of total) obtained information without considering the creditability of the source. Only one-third of the students searched for information specifically from educational and government websites (Fig. 1B).

### Limiting factors of OER access among students

The difficulty to read on digital screens for a longer period and lack of time during academic hours were the main constraints for participating student to use OER. However, the difficulty to read on digital screens is a constrain not limited to OER. Reading from digital devices can disturb the emotional feelings of users as well [58]. Therefore, teachers and administrators need to consider these constraints when proposing the use of OER to students (Table 3).

### SM usage among students

Around 90% of students used SM for academic purposes (Table 2) and this was similar to the use of SM among undergraduate Informatics students in Malaysia [59]. However, previous studies in Nigeria (4%) [60] and Kuwait (30%) showed a low SM usage for learning purposes [61]. Furthermore, SM usage in our study was not different based on gender or future postgraduate study plans (Table 2) as shown in previous studies [62, 63].

Facebook was the most popularly used SM platform among our participants (Table 4) as previously reported in the United Kingdom [64]. Around 80% of students used Facebook and more than half used YouTube and Google + services for their education [65].

Therefore, educators and organizations can use this high SM usage to connect with students and to deliver educational materials to students as previously highlighted [66–68].

Students showed a comparatively higher daily SM access than OER. Further, SM can be used to facilitate group interactions among the students [69, 70]. Therefore, frequent user engagement in SM and group interactions can be used to facilitate student learning activities.

### Interactive features in SM

Knowledge sharing and discussion are important features in SM [71, 72]. Most of the students joined education groups within SM and followed medical-related sites, pages and people (Table 5). This was higher when compared to participation in other medical forums found on the Web, thus indicating the SM is a good tool to encourage engagement in educational activities and group activities [37, 69, 70]. The first-year students showed higher engagement in academic discussions and sharing information compared to the final year students (Table 6). This may be because the younger students are computer-savvy than the older students [68, 73]. Previous studies have shown that low IT literacy can have a negative effect on senior students when promoting SM [70]. Further, senior students undergo more clinical training in hospital settings than junior students and they have less opportunity to use computer and Internet facilities at the Faculty.

### Limiting factors of SM access among students

Web 2.0 has permitted students to access information in unprecedented volume [48]. The main obstacles faced by students were the overload of information and the unreliability of shared materials in SM [60, 74]. A considerable portion of students (31%) in the present study (Table 5) reported that SM disturbed their academic work [57, 60, 70]. A previous study in Sri Lanka has reported that heavy Facebook usage was related to low grades of university students [75].

### The importance of information literacy in the learning process

Connectivism theory highlights that the ability to draw distinctions between important and unimportant information is vital and the ability to make decisions on the information is critical in the learning process [32]. Therefore, students need to identify the resources that provide important and reliable information to enhance critical thinking in the learning process. Therefore, improving IL skills is important to facilitate the learning process in this digital era. This is not only relevant to medical education but also to all the students in higher education.

### Limitations

Following are the limitations of this study. The study was carried out only in a single institution of the country. This may affect the generalizability of the results although the Faculty receives students from the entire country. This study analyzed the self-reported data which warrants caution when interpreting. A higher percentage of female students responded due to the adaptation of stratified random sampling method which can affect the overall usage patterns. The questionnaire was mainly based on a previous study conducted in Sri Lanka as a result, only a selected number of OER and SM platforms were assessed in this study. Therefore, the usage reported in this study may not provide information on the use of all OER and SM platforms available.

## Conclusion

The study revealed that most students used OER and SM for education. Wiki sites were the most used OER, and Facebook was the most used SM platform. Daily educational access was high in SM and information overload was the main obstacle in it. Students were concerned about concentration difficulties when using SM [60, 75]. The ability to find reliable information was low among students [76]. Therefore, IL needs to be improved among students to help them obtain the maximum benefits of web-based technologies including OER and SM [48].

## Supplementary Information

**Additional file 1: Supplementary material 1.** Sample size calculation. Sample size calculation with formulae. Student Usage of Open Educational Resources and Social Media at a Sri Lanka Medical School. Samankumara Hettige, Eshani Dasanayaka and Dileepa Senajith Ediriweera.

**Additional file 2: Supplementary material 2.** Questionnaire items in this study. Questionnaire items used to collect data. Student Usage of Open Educational Resources and Social Media at a Sri Lanka Medical School. Samankumara Hettige, Eshani Dasanayaka and Dileepa Senajith Ediriweera.

## Data Availability

Data are available on request to the authors. The data that support the findings of this study are available from the corresponding author (Samankumara Hettige) on request.

## Abbreviations

OER: Open Educational Resources
SM: Social Media
IL: Information Literacy
