# Supplementary material for: Student usage of open educational resources and social media at a Sri Lanka Medical School
Source: BMC Med Educ. 2022 Jan 13;22:35. doi: 10.1186/s12909-022-03106-2 (PMC8756628; doi:10.1186/s12909-022-03106-2)
Supplement: Supplementary file 1 — Additional file 1: Supplementary material 1. Sample size calculation. Sample size calculation with formulae. Student Usage of Open Educational Resources and Social Media at a Sri Lanka Medical School. Samankumara Hettige, Eshani Dasanayaka and Dileepa Senajith Ediriweera. [file 12909_2022_3106_MOESM1_ESM.docx]

**Student Usage of Open Educational Resources and Social Media at a Sri Lanka Medical School**

Samankumara Hettige, Eshani Dasanayaka and Dileepa Senajith Ediriweera

**Supplementary material 1 -Sample size calculation**

Sample size was calculated using the following formula and the assumptions.

| Sample size = | Z ^2^* (p) * (1-p) |
| --- | --- |
|  |  |
|  | c ^2^ |

Where:

Z = Z value (i.e. Z=1.96 for 95% confidence level) 
p = percentage picking a choice, expressed as decimal (i.e. p=0.5)
c = confidence interval, expressed as decimal (i.e. c=0.05).

Hence, the sample size was calculated as 384. Additional 10% was added as non-response bias (384 + 38 = 422). There were 903 total students in the Faculty and the calculated sample size represented 47% of the population. Hence the sample size is more than 5% of the total population, finite population correction (FPC) was adopted to avoid the over sampling.

Fine population correction (FPC):

| New sample size  = | n_0_ |
| --- | --- |
|  |  |
|  | (1 + 1/N (n_0_ -1)) |

Where:

n_0_=Initial sample size (i.e. 422)

N=Population size (i.e. 903)

Revised sample size was calculated as 287.
