# Supplementary material for: Student usage of open educational resources and social media at a Sri Lanka Medical School
Source: BMC Med Educ. 2022 Jan 13;22:35. doi: 10.1186/s12909-022-03106-2 (PMC8756628; doi:10.1186/s12909-022-03106-2)
Supplement: Supplementary file 2 — Additional file 2: Supplementary material 2. Questionnaire items in this study. Questionnaire items used to collect data. Student Usage of Open Educational Resources and Social Media at a Sri Lanka Medical School. Samankumara Hettige, Eshani Dasanayaka and Dileepa Senajith Ediriweera. [file 12909_2022_3106_MOESM2_ESM.docx]

**Student Usage of Open Educational Resources and Social Media at a Sri Lanka Medical School**

Samankumara Hettige, Eshani Dasanayaka and Dileepa Senajith Ediriweera

**Supplementary material 2- Questionnaire items in this study**

Add √ mark in the appropriate cage.

| \| **1** \| **2** \| **3** \| **4** \| **5** \| \| --- \| --- \| --- \| --- \| --- \| \|  \|  \|  \|  \|  \|   **Academic year** | \| **Male** \| **Female** \| \| --- \| --- \| \|  \|  \|   **Gender** |
| --- | --- | --- | --- | --- | --- | --- | --- | --- | --- | --- | --- | --- | --- | --- | --- |
| **Last continues assessment results**   \| **A** \| **B** \| **C** \| **D** \| **E** \| \| --- \| --- \| --- \| --- \| --- \| \|  \|  \|  \|  \|  \| | **Are you planning to do postgraduate studies?**   \| Yes \| No \| Not sure \| \| --- \| --- \| --- \| |

**Part 01-Open Educational Resources**

1. Do you use online resources for academic learning?

| 1.  Daily | 2.  More than once a week | 3.  More than once a month | 4.  Less than once a month | 5.  Never |
| --- | --- | --- | --- | --- |
|  |  |  |  |  |

1. If you use online resources, what is the the **most relevant** answer according to you
   1. Availability of descriptive and detailed information.
   2. Available at any time, any where
   3. Updated information can be found
   4. Can search relevant information easily
   5. Other …………………………………
2. How do you find information on web **most frequently**?
   1. Looking for results without considering the site (e.g. surf through the results)
   2. Searching specific sites (e.g. .edu, .gov, nih.gov)

| Yes | No |
| --- | --- |

1. Do you use wikis as source of information?

(e.g.: Wikipedia, Medpedia, AskDrWiki)

| 1. How would you rank the scientific validity of the contents in wikis according to you?  \| 1.  I don’t know \| 2  No validity \| 3.  Validity is less \| 4.  Acceptable level validity \| 5.  High Validity \| \| --- \| --- \| --- \| --- \| --- \| \|  \|  \|  \|  \|  \|  1. What are the online libraries/data bases you access most?    - 1. Hinari      2. Medline      3. Ebsco Host  \| Yes \| No \| \| --- \| --- \|  - - 1. Sage     2. Other(Please mention ...............................  \| Yes \| No \| \| --- \| --- \|  1. Do you access e journals for academic learning? 2. Do you access e journals provided by the Faculty of Medicine?  \| Yes \| No \| \| --- \| --- \|  1. Do you actively participate any medical forums?  \| Yes \| No \| \| --- \| --- \|  1. Do you use slide share as a learning tool? 2. If yes, Frequency of using SlideShare?  \| 1.  Daily \| 2.  More than once a week \| 3.  More than once a month \| 4.  Less than once a month \| \| --- \| --- \| --- \| --- \| \|  \|  \|  \|  \| |
| --- | --- | --- | --- | --- | --- | --- | --- | --- | --- | --- | --- | --- | --- | --- | --- | --- | --- | --- | --- | --- | --- | --- | --- | --- | --- | --- |

**Part 2 – Social Media**

1. Specify your social media usage for educational purposes as below.

(Mark weather you have an account or not and then the frequency of using them to academic learning)

| Social Media | I have an account | Frequency of usage for educational purposes | | | |
| --- | --- | --- | --- | --- | --- |
|  |  | Daily | Once a week | Once a month | Never |
| Facebook | \| Yes \| No \| \| --- \| --- \| |  |  |  |  |
| Google+ | \| Yes \| No \| \| --- \| --- \| |  |  |  |  |
| YouTube | \| Yes \| No \| \| --- \| --- \| |  |  |  |  |
| Twitter | \| Yes \| No \| \| --- \| --- \| |  |  |  |  |
| Blogs | \| Yes \| No \| \| --- \| --- \| |  |  |  |  |
| Google Docs | \| Yes \| No \| \| --- \| --- \| |  |  |  |  |
| Other………………… |  |  |  |  |  |
| Other………………… |  |  |  |  |  |
| Other………………… |  |  |  |  |  |

1. Have you joined any educational groups in social media?

(e.g. User group for the batch in Facebook /google+)

| Yes | No |
| --- | --- |

| Yes | No |
| --- | --- |

13)Do you follow medical related sites, pages, and people through your social media accounts? (Facebook/twitter/etc)

14))Do you have friends from other universities (Local or foreign) and have academic discussion or information sharing groups through your social media?

| Yes | No |
| --- | --- |

16)Which one of the below **mostly effect or limit** using Online resources as education tool according to you? (**Mark only one)**

- 1. Hard to read the screen for longer period
  2. No time during academic hours
  3. I don’t trust the content in the web
  4. I cannot find what I really need
  5. I don’t have a PC/laptop/smart phone /Internet

| 17) Which one of the below **mostly effect/limit** using social media as an education tool according to you? (**Mark only one**)   1. It fragment/disturb concentration on academics 2. Contains excess of unwanted information 3. I do not trust the content shared by others 4. I don’t have a PC/laptop/smart phone and internet |
| --- |
